# Analysis of clinical characteristics and factors influencing herpes simplex virus keratitis

**DOI:** 10.3389/fmed.2023.1267783

**Published:** 2024-01-16

**Authors:** Shuai Zhang, Jinhua Mi, Shengmei Ge, Guoqiang Wang, Zhongyou Zhou, Yantao Zhao, Yan Zhao

**Affiliations:** ^1^Department of Ophthalmology, The First Hospital of Hebei Medical University, Shijiazhuang, Hebei, China; ^2^Intensive Care Medicine, The Forth Hospital of Hebei Medical University, Shijiazhuang, Hebei, China

**Keywords:** herpes simplex keratitis, clinical characteristics, influencing factors, corneal opacity, eye pain

## Abstract

**Objective:**

To investigate the clinical characteristics and factors associated with herpes simplex virus keratitis.

**Methods:**

Patients with herpes simplex virus keratitis who came to our hospital from January 2018 to June 2022 were selected and divided into a good prognosis group and a poor prognosis group according to their prognosis. The clinical data of the two groups were compared, and univariate/multivariate logistic regression was used to analyze the factors influencing the poor prognosis of herpes simplex virus keratitis.

**Results:**

A one-way analysis of variance showed that, compared with the good prognosis group, the poor prognosis group had more elderly patients and a longer course of disease, and the difference was statistically significant (*p* < 0.05). There were significant differences in the types of patients between the two groups (*p* < 0.05). Univariate logistic regression analysis also showed that age (≥65 years) (OR: 1.557, 95%CI: 1.081–2.183, *p* < 0.05), course of disease (> 7 months) (OR: 1.303, 95%CI: 1.003–1.829, *p* < 0.05), epithelial type (OR: 2.321, 95%CI: 1.198–4.321, *p* < 0.05), and stromal type (OR: 2.536, 95%CI: 1.672–3.871, p < 0.05) were risk factors for poor prognosis. Multivariate logistic regression analysis showed that age (≥65 years) (OR: 1.656, 95%CI: 1.168–2.357, *p* < 0.05) and course of disease (> 7 months) (OR: 1.461, 95%CI: 1.031–2.001, p < 0.05) were independent risk factors for the prognosis of herpes simplex keratitis.

**Conclusion:**

The clinical symptoms of herpes simplex virus keratitis include corneal opacity, corneal posterior elastic layer folds, corneal infiltration, posterior corneal mass, corneal edema, and ocular pain. Age and course of disease are important factors in the prognosis of herpes simplex virus keratitis.

## 1 Introduction

Herpes simplex keratitis is a relatively common and serious infectious corneal disease in ophthalmology, and its incidence and blindness rate are increasing (1, 2)^.^ Herpes simplex virus keratitis is mainly caused by herpes simplex virus (HSV) infection, which is mainly observed in the clinic as HSV-1. Clinically, it can be divided into recurrent infection and primary infection and can be divided into disk, map, dendritic, and spot depending on the morphological manifestations of the lesions. Depending on the site of invasion, it can be further classified as a superficial layer or a deep layer. The superficial layer is mainly divided into common clinical epithelial types, and its clinicopathological morphology is mainly dendritic and punctiform. Its pathogenesis is that HSV directly infects the epithelial cells of the cornea, and its proliferation in the cells causes the degeneration and necrosis of corneal epithelial cells and the formation of epithelial defects (3). Herpes simplex virus keratitis is characterized by prolonged and difficult-to-cure, repeated relapses. This condition, if therapy is delayed or negligent, will seriously affect vision and may even lead to blindness. Currently, there is no specific drug for the treatment of herpes simplex virus keratitis in clinics, which is usually treated with glucocorticoid and antiviral drugs. However, due to the adverse reactions of hormone drugs and cell tinning of antiviral drugs, their clinical application has certain limitations, and their therapeutic effect is not good (4, 5). Therefore, it is the focus of clinical research to actively analyze the clinical characteristics and influencing factors of patients with herpes simplex virus keratitis and give targeted treatment to improve the prognosis of patients. Based on this, the clinical characteristics and influencing factors of patients with herpes simplex virus keratitis were discussed in this study in order to provide evidence for the clinical prevention and treatment of herpes simplex virus keratitis.

## 2 Patients and methods

1.1 Subjects: A total of 198 patients with herpes simplex virus keratitis admitted to our hospital from January 2018 to June 2022 were selected and divided into 166 patients with a good prognosis and 32 patients with a poor prognosis according to their prognostic status. Good prognosis group: 86 men, 80 women. Poor prognosis group: 17 men, 15 women. There was no significant difference in general information between the two groups (*p* < 0.05). The research protocol was formulated in accordance with the relevant requirements of the World Medical Association’s Declaration of Helsinki.

### 2.1 Inclusion and exclusion criteria

Inclusion criteria: Diagnosis of herpes simplex virus keratitis according to the diagnostic criteria in the Guidelines for the Diagnosis and Treatment of Eye Diseases by the American Academy of Ophthalmology (3rd Edition) (6): accompanied by a foreign body, irritation, redness, blurred vision, excessive tearing, photophobia, pain, and other symptoms; there are typical corneal signs, such as map and dendritic corneal ulcers, corneal stroma disk infiltration or edema, and subcutaneous deposits in the focal area of the cornea. The subjects should have a history of recurrent attacks and monocular disease and often appear to have fever, fatigue, or mental stress before the onset of the disease. The subjects should have tested positive in laboratory tests such as HSV-1 antibody or viral PCR analysis, and these specimens should have been obtained from the diseased site of the cornea by an ophthalmologist using a sterile procedure. Those who have not received antiviral, hormone, and other related treatments within 1 month; those with normal liver and kidney function. Exclusion criteria: patients with cataracts, iritis, glaucoma, and other eye diseases; patients with hematological diseases; those with complications of malignant tumors; those with immune system dysfunction; those with coagulation dysfunction; and finally, those who did not want to participate in this study.

### 2.2 Methods

Ganciclovir eye gel for external use (Hubei Keyi Pharmaceutical Co., LTD., 181,204/T) was dropped into the conjunctival sac, 1 drop each time, 4 times a day, for 3 weeks; Interferon alpha-2b eye drops were dropped into the conjunctival sac of the affected eye, 1–2 drops each time, 6 times a day for 3 weeks, with the eye closed for 1–2 min after application; and Acyclovir tablets (Shanghai No.6 Pharmaceutical Factory, batch number 971101), 200 mg each time, 5 times a day for 10 d.

### 2.3 Observation indicators

1.4.1 Clinical data: age, gender, BMI, course of disease (time from the patient’s first visit to this visit), type, drinking, smoking, corneal opacity, corneal posterior elastic layer folds, corneal infiltration, posterior corneal mass, corneal edema, and related complications of all subjects were collected.

Types: All subjects were classified according to the examination results of saccular lamp microscopy and corneal fluorescein staining: (1) Neurotype: Neurotrophic keratopathy was manifested; the punctured epithelial cells of the cornea were seen as erosion, then developed into persistent epithelial injury, an epithelial defect with smooth edges, gray thickening, and regular ulcerous lesions. (2) Epithelial type: presents with epithelial infectious keratitis with punctate microvesicles, dendritic corneal ulcers, or map-like corneal ulcers in the epithelium. (3) Stromal keratitis: presents as stromal keratitis, which can be further divided into necrotizing and non-necrotizing. Necrotizing stromal keratitis is usually characterized by corneal ulcers and dense stromal infiltrating lesions accompanied by epithelial defects. Non-necrotizing stromal keratitis may present as focal, multifocal, or diffuse infiltration without the formation of ulcers. (4) Endothelial type: presents with corneal endotheliitis (corneal endotheliitis is a specific inflammatory reaction of corneal endothelial cells, which is characterized by corneal edema, keratic deposits (KP), a mild inflammatory reaction in the anterior chamber, and corneal endothelial cell damage) and iritis (stromal edema may be disciform, linear, or diffuse). (5) Mixed type: the above two types exist simultaneously.

1.4.2 Prognosis: the prognosis was determined according to the follow-up after treatment (6); Cure: symptoms such as ocular congestion and pain disappear; fluorescent staining is positive; corneal ulcer disappears; no corneal edema; the HSV-1 antibody test or the virus PCR detection test are negative; Effective: All stimuli and symptoms are alleviated; the area of the corneal ulcer is reduced; the amniotic fat of the corneal wall is reduced; and the HSV-1 antibody or the virus PCR are detected and analyzed. Ineffective: none of the above symptoms have changed or appear to be worsening, and the HSV-1 antibody test or the virus PCR test were positive. Effective rate = cure rate + effective rate. If it is effective, the prognosis is good; if it is ineffective, the prognosis is poor.

### 2.4 Statistical analysis

SPSS 21.0 software was used to analyze the data and create an Excel database. The measurement data conforming to the normal distribution were expressed as ±s. The overall comparison of the data in each group was analyzed by one-way variance, and the comparison between and within groups was performed by the LSD method. The statistical data were expressed as a rate (%) and compared by the chi-square test. Univariate/multivariate logistic regression analysis was used to analyze the factors influencing the poor prognosis of herpes simplex virus keratitis, and *p* < 0.05 was considered a significant difference.

## 3 Results

### 3.1 Comparison of general data between the two groups

After treatment, the efficacy rate of all subjects in this study was 83.83% (166/198). There was no significant difference between the two groups in terms of gender, BMI, alcohol consumption, smoking, corneal opacity, posterior elastic corneal layer fold, corneal infiltration, posterior corneal mass, corneal edema, eye pain, complications, and other aspects (*p* > 0.05). When compared with the course of disease and classification, the differences were statistically significant (*p* < 0.05), as shown in Table 1.

**Table 1:** Comparing the general data of the two groups.

Project	Good prognosis group (*n* = 166)	Poor prognosis group (*n* = 32)	χ^2^/t	*p*
Gender (*n*%)			0.019	0.891
Male subjects	86 (51.8%)	17 (53.1%)		
Female subjects	80 (48.2%)	15 (46.9%)		
Age (*n*%)			4.807	0.028
≥65	79(47.6%)	22(68.8%)		
<65	87(52.4%)	10(31.2%)		
BMI(kg/m^2^)	24.87 ± 3.23	24.78 ± 3.38	0.143	0.886
Course of disease (months)	4.03 ± 0.69	9.03 ± 0.73	−37.184	<0.001
Types (*n*%)			11.637	0.020
Epithelial type	53(31.9%)	17(53.1%)		
Neurotype	50(30.1%)	1(3.1%)		
Stromal type	50(30.1%)	12(37.5%)		
Endotheliform	5(3.0%)	1(3.1%)		
Mixed type	8(4.8%)	1(3.1%)		
Alcohol consumption (*n*%)	20(12.0%)	4(12.5%)	0.005	0.943
Smoking (*n*%)	16(9.6%)	3(9.4)	0.002	0.963
Clinical symptoms (n%)			0.499	0.992
Corneal opacity	130(78.3%)	29(90.6%)	2.571	0.109
Fold of corneal posterior elastic layer	135(81.3%)	27(84.4%)	0.168	0.682
Corneal infiltration	121(72.9%)	28(87.5%)	3.074	0.080
Posterior corneal deposit	127(76.5%)	29(90.6%)	3.200	0.074
Corneal edema	122(73.5%)	28(87.5%)	2.866	0.090
Ocular pain	125(75.3%)	29(90.6%)	3.645	0.056
Complications (*n*)				
Diabetes	17(10.2%)	6(18.8%)	1.892	0.169
Hypertension	12(7.2%)	4(12.5%)	1.004	0.316

### 3.2 Single-factor logistic regression analysis was used to analyze the factors influencing the poor prognosis of herpes simplex virus keratitis

Independent variables were set as indicators with differences in comparison in general data, including gender, BMI, course of the disease, classification, alcohol consumption, smoking, corneal opacity, corneal posterior elastic folds, corneal infiltration, posterior corneal mass, corneal edema, eye pain, and complications. Dependent variables were the poor prognosis of herpes simplex keratitis, and univariate logistic regression analysis was performed. The results showed that the factors affecting the prognosis of herpes simplex virus keratitis were age (≥65 years), course of disease (> 7 months), epithelial type, and stromal type, as shown in Table 2.

**Table 2:** Single-factor logistic regression analysis of poor prognostic factors in herpes simplex virus keratitis.

Variable	*β*	SE	Waldχ2 value	OR(95%CI)	*p* value
Gender (Male subjects)	1.321	0.701	3.498	0.987(0.061–1.089)	0.063
Age (n)(≥65)	0.573	0.193	6.528	1.557(1.081–2.183)	0.001
BMI(24.00 kg/m^2^)	0.109	0.281	0.132	1.128(0.897–1.328)	0.573
Course of disease (> 7 months)	0.283	0.202	6.359	1.303(1.003–1.829)	0.007
Types					
Epithelial type	0.969	0.397	5.721	2.321(1.198–4.321)	0.013
Neurotype	0.868	0.713	1.516	2.282(0.581–6.756)	0.213
Stromal type	0.898	0.276	11.823	2.536(1.672–3.871)	<0.001
Endotheliform	0.265	0.693	1.456	2.189(1.002–3.492)	0.224
Mixed type	3.804	2.009	3.321	3.291(1.204–7.023)	0.069
Alcohol consumption	0.259	0.243	1.069	0.989(0.456–1.342)	0.309
Smoking	0.098	0.162	0.337	0.919(0.076–1.321)	0.567
Corneal opacity	0.083	0.239	0.317	1.083(0.678–1.719)	0.739
Fold of corneal posterior elastic layer	0.152	0.187	0.847	1.193(0.812–1.629)	0.391
Corneal infiltration	0.048	0.256	0.209	1.059(0.628–1.769)	0.834
Posterior corneal deposit	0.169	0.349	0.472	1.183(0.578–2.365)	0.638
Corneal edema	0.067	0.269	0.062	1.067(0.629–1.819)	0.819
Eye pain	0.457	0.273	2.731	1.345(0.909–2.312)	0.091
Complications (*n*)					
Diabetes	0.339	0.689	0.217	1.323(0.238–4.871)	0.628
Hypertension	0.239	0.419	0.329	1.238(0.548–2.781)	0.567

### 3.3 Multivariate logistic regression analysis was used to analyze the factors influencing the poor prognosis of herpes simplex virus keratitis

Independent variables were set as indicators with differences in general data, including age, course of disease, epithelial type, and stromal type. Dependent variables were the poor prognosis of herpes simplex virus keratitis. Multivariate logistic regression analysis showed that the main factors influencing the poor prognosis of herpes simplex virus keratitis were age and course of disease, as shown in Table 3.

**Table 3:** Multivariate logistic regression analysis of factors influencing poor prognosis in herpes simplex virus keratitis.

Variable	*β*	SE	Waldχ2value	OR(95%CI)	*p* value
Age (≥65 years old)	0.528	0.178	2.839	1.656(1.168–2.357)	0.003
Course of disease (> 7 months)	0.383	0.176	2.452	1.461(1.031–2.001)	0.029
Epithelial type	0.059	0.276	0.203	1.067(0.782–1.783)	0.839
Stromal type	0.156	0.181	0.845	1.162(0.819–1.628)	0.392

## 4 Discussion

Herpes simplex keratitis is a major corneal disease that causes visual damage worldwide. Studies have shown that 90% of U.S. adults (7) are positive for herpes simplex virus seroantigen, but only 20–30% of them have relevant clinical symptoms, most of which manifest as oral sores, and relatively few of them have ocular manifestations. According to relevant studies (8), the annual incidence of herpes simplex virus keratitis is 5.9 to 20.7 per million people, which may reach 149 per million people in developed countries. Herpes simplex virus, whose plasmid size is about 180 nm, is a kind of DNA virus that can infect humans. It can be divided into type I (HSV-1) and type 2 (HSV-2), and the majority of eye infections are caused by type 1 (9). Due to the long course of the disease and the high recurrence rate, the disease will have a negative impact on the life, work, and study of patients, seriously reduce their quality of life, and may even lead to blindness in some cases because of the treatment not being done in time. Therefore, more efforts should be made to prevent and treat herpes simplex virus keratitis.

Currently, there is no specific agent for the clinical treatment of herpes simplex virus keratitis, and antiviral therapy is the main treatment method, depending on the herpes virus causing the disease (10, 11)^.^ In the clinical antiviral treatment of herpes simplex virus keratitis, commonly used drugs include acyclovir, ganciclovir eye fluid, or eye gel. Ganciclovir eye gel is a nucleoside antiviral drug, and its mechanism of action in the treatment of herpes simplex virus keratitis is as follows: after the drug enters human cells, it phosphorylates ganciclovir monophosphate to ganciclovir triphosphate by cell kinase, thus inhibiting the replication of herpes virus (12). The practice has confirmed (13) that ganciclovir eye gel has the effect of less irritation, longer duration of efficacy, and can increase the local concentration and duration of action, thus promoting the improvement of eye irritation symptoms. However, the easy access to ganciclovir eye gel has certain limitations. For example, repeated use can easily induce drug resistance in patients, leading to unsatisfactory treatment efficacy. Recombinant human interferon alpha-2b eye drops, indicated for the treatment of herpes simplex virus keratitis, have spectroviral activity, can quickly reach the site of the lesion, protect target cells, and prevent the spread of the virus, effectively killing it. The combined use of ganciclovir eye gel and recombinant human interferon alpha-2b eye drops can not only inhibit the replication of the virus, but also enhance the body’s immunity, with better long-term effects while improving efficacy (14). After treatment with ganciclovir eye gel combined with interferon alpha-2b eye drops, the efficacy rate of the subjects in this study reached 83.83% (166/198), although it was lower than that of previous studies, which may be caused by different subjects enrolled in the study.

Herpes simplex keratitis mostly occurs in one eye, with binocular disease accounting for only 1.3–12% of cases, and is mostly caused by reduced immune function (15). In this study, seven patients (3.54% (7/198)) developed binocular disease, which is consistent with the above research results. Previous studies (16) indicated that the incidence of herpes simplex keratitis was similar in men and women. Multiple studies (17) have indicated that the male-to-female ratio of patients with herpes simplex virus keratitis ranges from 0.83 to 1.4. The results of this study showed that the ratio of female-to-male patients with herpes simplex virus keratitis was 1.08 to 1, which is consistent with the above findings. Herpes simplex keratitis can cause inflammatory reactions in various layers of the cornea. In the past, depending on the anatomical site of the lesion, herpes simplex keratitis was clinically classified as endothelial (manifested as endokeratitis), epithelial (manifested as epithelial infectious keratitis), stromal (manifested as stromal keratitis), neurotrophic keratitis (manifested as neurotrophic keratitis), and mixed type (18). A French study (19) indicated that the epithelial type was the most common type in patients with recurrent herpes simplex virus keratitis. In this study, the classification comparison between the good prognosis group and the poor prognosis group (*p* < 0.05) was consistent with the above research results. In patients with a poor prognosis of herpes simplex virus keratitis, epithelial and stromal types were more common, while neural, endothelial, and mixed types were relatively rare. A study by Wang Li pointed out that the clinical symptoms of severe herpes simplex virus keratitis include corneal opacity, corneal posterior elastic layer folds, corneal infiltration, posterior corneal mass, corneal edema, eye pain, and so on. In this study, the clinical symptoms of herpes simplex virus keratitis included corneal opacity, corneal posterior elastic layer folds, corneal infiltration, posterior corneal mass, and corneal edema, which were also consistent with the results of previous studies.

The findings of this study showed that the factors influencing the poor prognosis of herpes simplex virus keratitis were age (≥65 years), course of disease (> 7 months), and epithelial and stromal type. The results of multivariate logistic regression analysis showed that the main factors affecting the prognosis of herpes simplex virus keratitis were age and course of disease, suggesting that age and course of disease are important factors affecting the prognosis of herpes simplex virus keratitis. The reason may be that the older the patient is, the more the physical function of the patient will decline; the resistance to the latent virus will also decrease, which may be complicated by a variety of underlying conditions (20). In addition, the longer the course of the disease, the more severe the condition may be, and the more difficult the treatment of severe cases, the more difficult the recovery - hence the poor prognosis.

In summary, the main clinical symptoms of herpes simplex virus keratitis include corneal opacity, corneal posterior elastic layer folds, corneal infiltration, posterior corneal mass, corneal edema, eye pain, and so on. Age and course of disease are important factors affecting the prognosis of the disease. However, this study also has many shortcomings. At present, there is no clinical analysis of the clinical characteristics and risk factors of herpes simplex virus keratitis, and the sample size included in this study is small. Coupled with the short follow-up time, this means that the sample size should be further expanded and the follow-up time should be extended so as to further confirm the results of this study.

## Data availability statement

The original contributions presented in the study are included in the article/supplementary material, further inquiries can be directed to the corresponding authors.

## Ethics statement

The studies involving humans were approved by Ethics Committee of The First Hospital of Hebei Medical University. The studies were conducted in accordance with the local legislation and institutional requirements. The participants provided their written informed consent to participate in this study.

## Author contributions

SZ: Conceptualization, Data curation, Formal analysis, Methodology, Writing – original draft. JM: Conceptualization, Data curation, Formal analysis. SG: Conceptualization, Data curation, Formal analysis, Writing – original draft, Writing – review & editing. GW: Data curation, Formal Analysis, Writing – review & editing. ZZ: Conceptualization, Data curation, Formal analysis, Methodology, Writing – review & editing. YantZ: Conceptualization, Data curation, Formal analysis, Writing – review & editing. YanZ: Conceptualization, Data curation, Formal analysis, Investigation, Writing – review & editing.
